# Intermittents and Remedies

**Published:** 1856-01

**Authors:** A. E. Goodwin

**Affiliations:** Rockford, Ill.


[WE insert the following Letter with pleasure, as we do almost everything calculated to draw attention to our indigenious Materia Medica.—EDITOR.]

ARTICLE III.—*Intermittents and remedies.*

DOCT. DAVIS,—Dear Sir,—It strikes me that in writing for a Medical Journal, our object should be to communicate some important facts relating to the *Practice of Medicine.*

I have endeavored in this article to fulfil this principle. I take the liberty to send it to you to dispose of as you think best.

Should you deem it possessing sufficient merit to be entitled a place among the pages of your Journal, it will gratify the writer, whose object has been to accomplish good to the medical fraternity, and hope its perusal will suffice to induce my brethren to ponder on the importance of the subject.

Ever since the middle of the seventeenth century, *Cinchona* has been the great remedy for intermittents. Its use has been almost universal as the specific, the *sine qua non* for miasmatic fevers or periodics.

Its use has grown into a *mania* among the people and profession. It is quinine, first and last—the *alpha* and *omega* in treatment.

However, the last few years, a chosen few have been awakened to a consideration of the article, and some prejudices have arisen unfavorable to its merits.

And we are happy to record, that it is yielding to legitimate medicine and inductive philosophy. That persistence in error does not always continue to sway the world. I would not be understood as totally discarding quinine in medicine or in intermittents. But that it has been overrated, been rode as a *hobby*, and *hobbyism* I detest. That there are equally as good and better remedies than *Cinchona*, or its *alkaloids*, for *fever* and *ague*, is what I would inculcate; that this is not the specific *par excellence;* that we have indigenous remedies far preferable as a curative, as feasible preparations, and at less expense. Quinine will certainly stop the chill; but sure as *fate*, it will again return upon slight exposure. In primary agues, it does very well, but in secondary protracted cases, other remedies are more certain and reliable.

Quinine is mobile in its effect, not imparting tone and vigor to the nervous centres; hence the great proclivity to relapses.

Let us not persist in its use, when our understandings are enlightened as to its inferiority in comparison with others. Wherever intermittents prevail, I have no doubt as to indigenous specifics. Let me furnish you a brief sketch of a few of the indigenous remedies of Illinois, equally as powerful and certain as quinine, dispensing with the trouble and expense beside of going to South America for an antidote, for we have it at home within our reach.

The *Hickory*, (*Juglans*) so common, is an excellent anti-periodic—the inner bark of either varieties in decoction. Hickory tea can be recommended as a safe and effectual remedy in cases of simple and confirmed agues.

The *Wild Crab Apple*, (*Pyrus malus*) is another, growing so plentifully in the West. A decoction of the root forms a good remedy for chills.

The *Poplar* (*Populus tremuloides*) is a superior remedy, and can be much relied upon.

Dogwood (*Cornus sericea*) is another powerful antidote, superseding by far the use of Cinchona, or any of its preparations, in cases of long standing.

The *Branch Willow*, (*Salix alba*) found along the margins of our creeks. The root or inner bark, in decoction, is an old and well-known remedy in fevers of the South, and cures chronics more effectually than quinine. Its alkaloid (*salicin*) is being much used.

The Chestnut, especially *Horse-Chestnut*, (*Æsculus Lippocastanum*) furnishes another remedy in the list.

Decoction of the corn blade and of corn meal will cure simple intermittents.

A decoction of Hickory, Willow, and Dogwood barks, one-half ounce each to a pint of water, taking a half glassful every two hours between the paroxysms, will cause them to succumb to its herculean powers! Or a decoction of any one single bark, one ounce to the pint of water, and dose one-half glassful, will fulfil its prescribed mission, that of curing as many, if not more, intermittents as *Cinchona* or *Quinine*.

If I were to select a foreign remedy for obstinate intermittents, it would be the *Faba sancti ignatii*—its extract, a half grain twice a-day. It is a reliable tonic, invigorating the nervous centres, thus preventing relapses. I have used it in my practice with beneficial results, and have not as yet known of its failure. It acts upon the emunctories, the skin, the kidneys, and bowels, increasing their functions—rendering it a very rational remedy for miasmatic fevers. I would be very much pleased to have some of my professional brethren test the above remedies, and report their experience. It is a subject which demands your consideration and investigation. Let us hear from our *Western Doctors*, that have dealt so much with the ague for the past few months.

Truly yours,

A. E. GOODWIN, M. D.

*Rockford, Ill., Dec.*, 1855.

